# Development and verification of an immune-related gene prognostic index for gastric cancer

**DOI:** 10.1038/s41598-022-20007-y

**Published:** 2022-09-20

**Authors:** Chen Zhang, Tao Liu, Jian Wang, JianTao Zhang

**Affiliations:** 1 General Surgery Center of the First Hospital of Jilin University, Changchun, Jilin People’s Republic of China; 2 Department of Spinal Surgery, Tianjin Hospital, Tianjin, People’s Republic of China

**Keywords:** Data processing, Databases, Genome informatics, Cancer genomics, Cancer microenvironment, Gastrointestinal cancer, Tumour biomarkers

## Abstract

Immune checkpoint inhibitor (ICI) therapy is an emerging and effective approach to the treatment of gastric cancer (GC). However, the low response rate of GC patients to ICI therapy is a major limitation of ICI therapy. We investigated the transcriptomic signature of immune genes in GC could provide a comprehensive understanding of the tumor microenvironment (TME) and identify a valuable biomarker to predict the response of GC patients receiving immunotherapy. We performed the weighted gene co-expression network analysis (WGCNA) to determine immune-related hub genes that differentially expressed in the GC dataset based on The Cancer Genome Atlas (TCGA). After that, univariate and multivariate Cox regression was performed to recognize prognostic genes associated with overall survival and to develop an immune-related gene prognostic index (IRGPI). Furthermore, we explored the possible correlation between IRGPI and immune cell infiltration and immunotherapy efficacy. Notably, IRGPI can predict the prognosis of GC patients, as well as the response to immunotherapy. IRGPI as an immune-related prognostic biomarker might bring some potential implications for immunotherapy strategies in GC.

## Introduction

Gastric cancer is the fifth most prevalent cancer type and the fourth leading cause of cancer death^1^. Immune-related gene (IRG) is identified through research as genes that are significantly associated with the individual or partial pathways of the immune response. IRG can be involved in the activation of immune cells, migration of immune cells, and release of inflammatory factors, and play an important role in the development and progression of cancer^2–4^. IRG can be used as a biomarker to predict the prognosis of cancer patients^5^. It has been shown that high expression of immune-related genes predicts a better prognosis for EBV-positive and EBV-negative gastric cancer patients^6^. Gastric cancer is being treated with surgery, chemotherapy, radiation therapy, and targeted therapy^7^. In recent years, immunotherapy has gained prominence as a viable cancer therapy. Immune checkpoint inhibitor (ICI) therapy, such as those targeting programmed death 1 (PD1), programmed death-ligand 1 (PD-L1), and cytotoxic T lymphocyte-associated protein 4 (CTLA4), has demonstrated considerable survival benefits. Inhibition of the PD-1/PD-L1 axis with ICIs (such as pembrolizumab and nivolumab) has been regarded as a prominent therapy strategy for advanced GC^8–14^. The main disadvantage of ICI therapy, however, is the limited response rate of patients to ICI treatment. Because tumor microenvironment (TME) and PD-L1 levels, among many others, affect ICI efficacy, few biomarkers are good predictors of patient prognosis^15^.

We discovered a marker to indicate the prognosis for GC in this investigation. In the GC transcriptome data, we searched for immune-related differential genes, identified hub genes using weighted gene co-expression network analysis (WGCNA) and CytoHubba plugin, and performed univariate Cox regression analysis to identify immune-related prognostic genes. Furthermore, multivariate Cox analysis identified five genes to create an immune-related gene prognostic index (IRGPI). Afterward, we investigated the molecular and immunological profile of the IRGPI group and compared it with TIDE (tumor immune dysfunction and exclusion) and TIS (tumor inflammatory signature). In addition, we compared the efficacy of IRGPI in the immunotherapy cohort and its prognostic value in immunotherapy patients. The findings indicate the IRGPI might be a potential biomarker.

## Methods and materials

### Collection of gastric cancer datasets

RNA-seq data and clinicopathological information of 407 GC samples, which include 375 tumor samples and 32 normal samples, and data on somatic mutation variation were obtained from The Cancer Genome Atlas (TCGA) (https://portal.gdc.cancer.gov/). The somatic mutation data was processed by the Maftool package and the TMB was obtained by Perl calculation script, which is the total number of somatic variants per million bases^16^. RNA-seq data and survival statistics for 300 GC samples (GSE62254) were also retrieved from the GEO database (https://www.ncbi.nlm.nih.gov/geo/)^17^. ImmPort (https://www.immport.org/shared/home) and InnateDB (https://www.innateDBdb.com/) databases were utilized to seek immune-related genes^18,19^. Standardized RNA expression data from the advanced gastric cancer anti-PD-1 therapy clinical cohort (PRJEB25780) were downloaded from Tumor Immune Dysfunction and Exclusion (http://tide.dfci.harvard.edu/), and immunotherapy clinical data were extracted from the manuscript^20,21^.

### Differentially expressed genes are identified

According to the criterion of |log2(FoldChange)|> 1 and false discovery rate (FDR) < 0.05, in TCGA-STAD dataset, the “limma” package of R was utilized to find differentially expressed genes between 375 tumor samples and 32 normal samples. After consideration in the context of the lists of immune-related genes collected from ImmPort and InnateDB, differentially expressed related immune genes were obtained. Then, WGCNA was exploited to determine the hub genes^22^. WGCNA could add phenotypic weight parameters in the process of constructing gene co-expression networks while using scale-free clustering and dynamic shear trees to optimize the classification for accurate and efficient analysis of the data. First, using the expression data, a similarity matrix was created by computing the correlation coefficient between genes. The similarity matrix was then converted into an adjacency matrix with a network type of signature and a soft threshold of β = 3 to indicate the degree of interaction between genes, and finally into a topology matrix with a topological overlap measure (TOM). The 1-TOM is employed as the gene clustering distance, and the modules are identified utilizing a dynamic pruning tree. Finally, by adjusting the merge threshold function, we are able to determine the number of relevant modules and extract the genes in the most differentiated modules. Furthermore, we used an open-source software platform STRING (https://string-db.org/) to build a PPI network of immune-associated DEGs. We selected the degree of interaction between proteins with a composite score greater than 0.9 and analyzed them with the use of Cytoscape. We then selected immune-associated hub genes using the cytoHubba plugin. Obtaining intersecting genes from modular genes and immune hub genes via the Draw Venn Diagram online tool (http://bioinformatics.psb.ugent.be/webtools/Venn/). The clusterProfiler package of R was used to evaluate genes using Gene Ontology (GO) and Kyoto Encyclopedia of Genes and Genomes (KEGG) analyses^23,24^.

### The immune-related gene prognostic index (IRGPI) was developed

To access prognosis-related immune genes, we used the survival package of R to do a univariate Cox regression analysis exploring the relationship between hub gene expression and overall survival (OS)^25^. We obtained 22 immune genes associated with prognosis. Among the 22 immune-related prognostic genes, multivariate Cox regression analysis was used to explore genes with substantial effects on OS and to calculate weights to construct prognostic models to develop IRGPI. In the Cox model, the IRGPI of each sample was determined by multiplying the expression levels of certain genes by their weight and then adding them together. K–M survival curves with log-rank testing were used to assess the predictive value of the IRGPI in both the TCGA and GEO cohorts. Univariate and multivariate Cox regression analyses were exploited to affirm IRGPI independent prognostic significance.

### In-depth analysis of molecular and immunological features in distinct IRGPI groups

The “limma” package of R was used to perform differential expression analysis on all genes to assess the samples with high and low IRGPI scores in the signaling pathway involved. The gene set enrichment analysis (GSEA) approach based on the KEGG gene sets was then used using the clusterProfiler package of R to find the signaling pathways in which the differentially expressed genes are implicated (p < 0.05). Using the R package Maftools, the gene mutation map was created in two IRGPI groups. To determine the immune characteristics of the 375 GC samples, we imported their expression data into CIBERSORT (https://cibersort.stanford.edu/) and perform 1000 iterations to determine the proportional proportions of the 22 immune cell types. The proportions of the 22 immune cells were then compared between the two IRGPI groups. We also explored the correlation between the IRGPI groups and the conventional immune subtypes. IRGPI score, PD-L1 expression, and TMB were all analyzed for correlation. To explore the IRGPI on patient immunotherapy, we evaluated two immunotherapy cohorts, IMvigor210 and PRJEB25780. In addition, we performed a time-dependent ROC curve analysis to obtain the area under the curve (AUC) and compared the prognostic value between IRGPI, TIDE, and TIS with the timeROC package of R. The TIDE score was obtained utilizing an internet tool (http://tide.dfci.atherard.edu/)^21^. TIS score was constructed as the mean of log2 scale-normalized expression of the 18 signature genes^26^.

### Statistical analysis

For the comparison of categorical variables, the Chi-square test was utilized. Independent t-tests were used to compare continuous variables between the two groups. A comparison of TIDE scores between groups was performed by the Wilcoxon test. The Kaplan–Meier survival analysis and log-rank test were applied to perform univariate survival analysis. Multivariate survival analysis was performed using the Cox regression model. A two-sided p value < 0.05 was deemed significant. The statistical analyses in this study were generated by R-4.0.4.

### Ethical approval

All methods in this study were performed in accordance with the relevant guidelines and regulations.

## Result

### Development of an immune-related gene prognostic index

**Figure 1:**
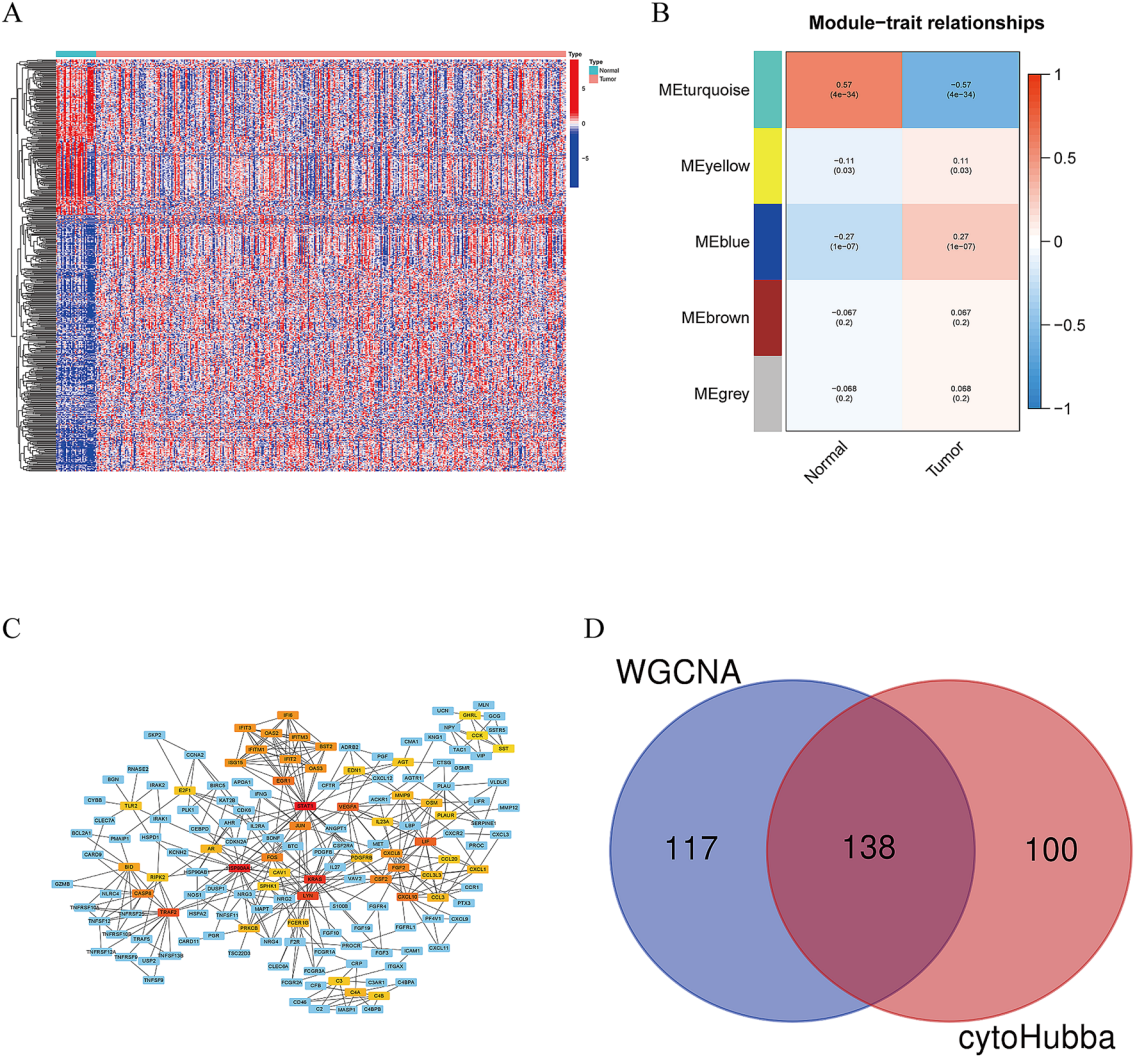
Selection of immune-related genes. (**A**) Heatmap showing differential immune-related gene expression between normal and gastric cancer tissues (**B**) Heatmap of the correlation between normal and tumor tissues on module genes. (**C**) PPI network diagram of immune hub genes. (**D**) Venn diagram shows the immune-related genes. [Figures created by R, version 4.0.4. STRING, https://string-db.org/; Cytoscape (https://cytoscape.org/; version 3.9.1)].

In differential expression analysis of 375 gastric cancer tissues and 32 normal samples in the TCGA cohort, we discovered 8832 DEGs, with 7497 genes upregulated and 1335 genes downregulated in the tumor samples compared to the normal samples. DEGs were overlapped with a list of immune-related genes derived from ImmPort and InnateDB, yielding 493 immune-related genes that are differentially expressed, with 309 upregulated and 184 downregulated (Fig. 1A; Table S1). To acquire immune-related hub genes, we first performed WGCNA on candidate genes (n = 493). The correlation coefficient was more than 0.8, and the logarithm log(k) of the node with connection K was negatively linked with the logarithm log (P (k)) of the node's probability. Based on the scale-free network, the optimal soft threshold power was 3. Refers to the average linkage hierarchical clustering and the optimal soft-thresholding power, five modules were determined (Fig. 1B, Fig. S1). We observed that the turquoise module was closely connected with GC tumors based on the Pearson correlation coefficient between a module and a sample characteristic, hence the genes in turquoise modules were chosen for further testing (n = 255). Secondly, we explore potential associations between immune genes, we performed protein–protein interaction (PPI) networks using the STRING, requiring interaction scores > 0.900. Then, we obtained 238 immune hub genes using the cytoHubba plugin and displayed the interaction network graph in Fig. 1C. We merged the turquoise module genes and immune hub genes to finally obtain 132 immune-related genes (Fig. 1D).

**Figure 2:**
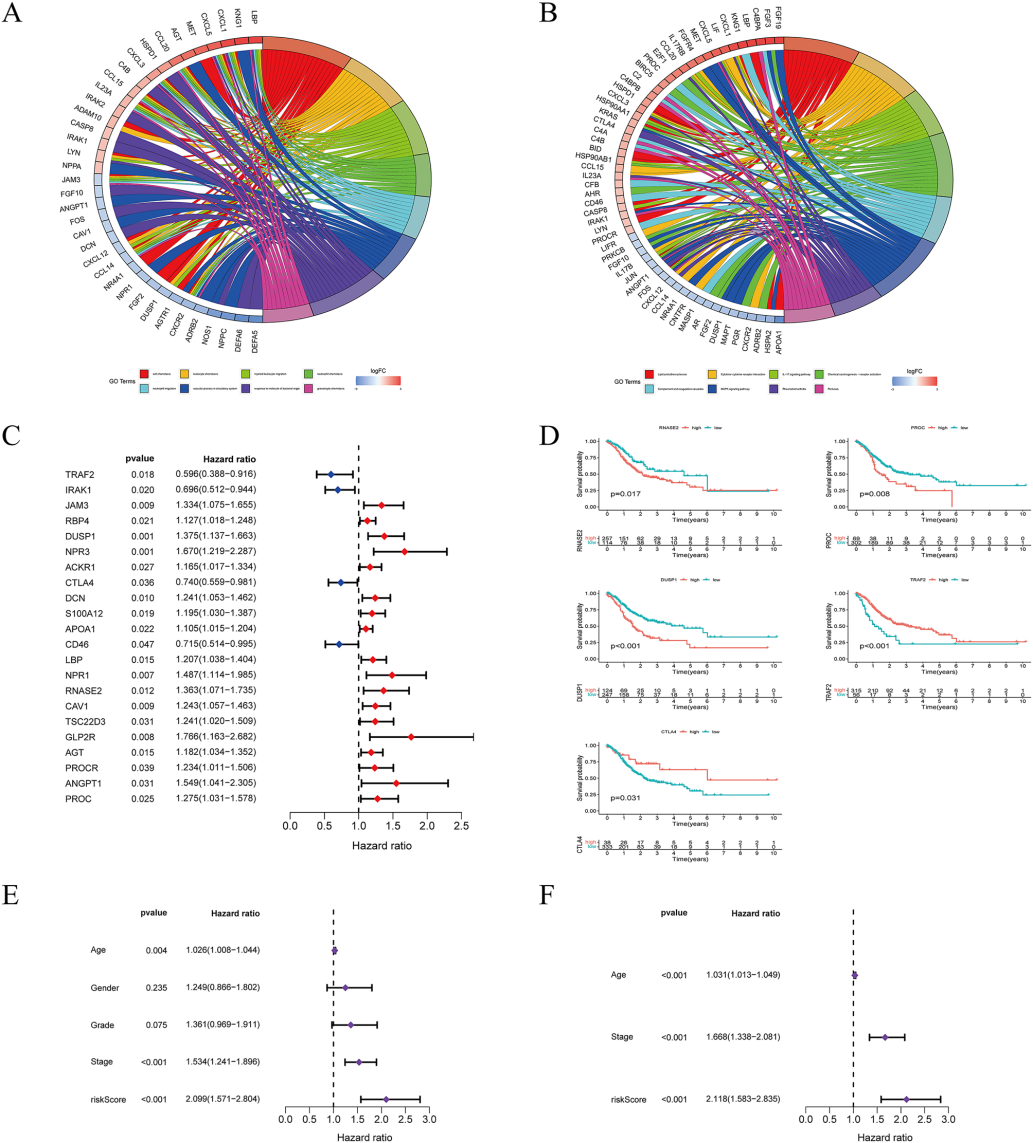
Screening of immune-related prognostic genes and development of IRGPI. (**A**) Gene Ontology (GO) analysis for the immune-related hub genes. (**B**) The Kyoto Encyclopedia of Genes and Genomes (KEGG) pathways for immune-related hub genes. (**C**) Univariate Cox analysis of 22 immune-related genes. (**D**) Kaplan–Meier survival analysis curves for five model genes. (**E**,**F**) Univariate and multivariate Cox regression analysis on IRGPI and other clinicopathologic factors. [Figures created by R, version 4.0.4.].

These genes were analyzed by GO and KEGG in Fig. 2A,B. According to Univariate Cox regression analysis, the expression of 22 immune-related prognosis genes has statistical significance in GC patients, as shown in Fig. 2C. Multivariate Cox regression analysis was conducted on 22 immune-related hub genes to uncover independent prominent prognostic genes. As shown in Fig. 2D, five genes (*TRAF2, CTLA4, DUSP1, PROC,* and *RNASE2*) substantially impact the OS of GC patients. Next, we developed a prognostic index by using IRGPI formula = RNASE2*(0.3361) + PROC*(0.3254) + CTLA4*(-0.3391) + DUSP1*(0.2699) + *TRAF2* *(− 0.3576). The IRGPI and other clinicopathologic factors were submitted to a univariate Cox regression analysis, which confirmed that the IRGPI, clinical stage, and age were significant determinants in the prognosis of GC (Fig. 2E). Furthermore, after controlling for other clinicopathologic characteristics, multivariate Cox regression analysis indicated that IRGPI was an independent predictive factor (Fig. 2F). We also investigated the association between IRGPI and clinical characteristics (Fig S2). We found that patients with gastric cancer (age ≤ 65) had higher risk score. In addition, there was no statistically significant difference between IRGPI and tumor stage.

### Survival outcomes and molecular characterization of different IRGPI groups

**Figure 3:**
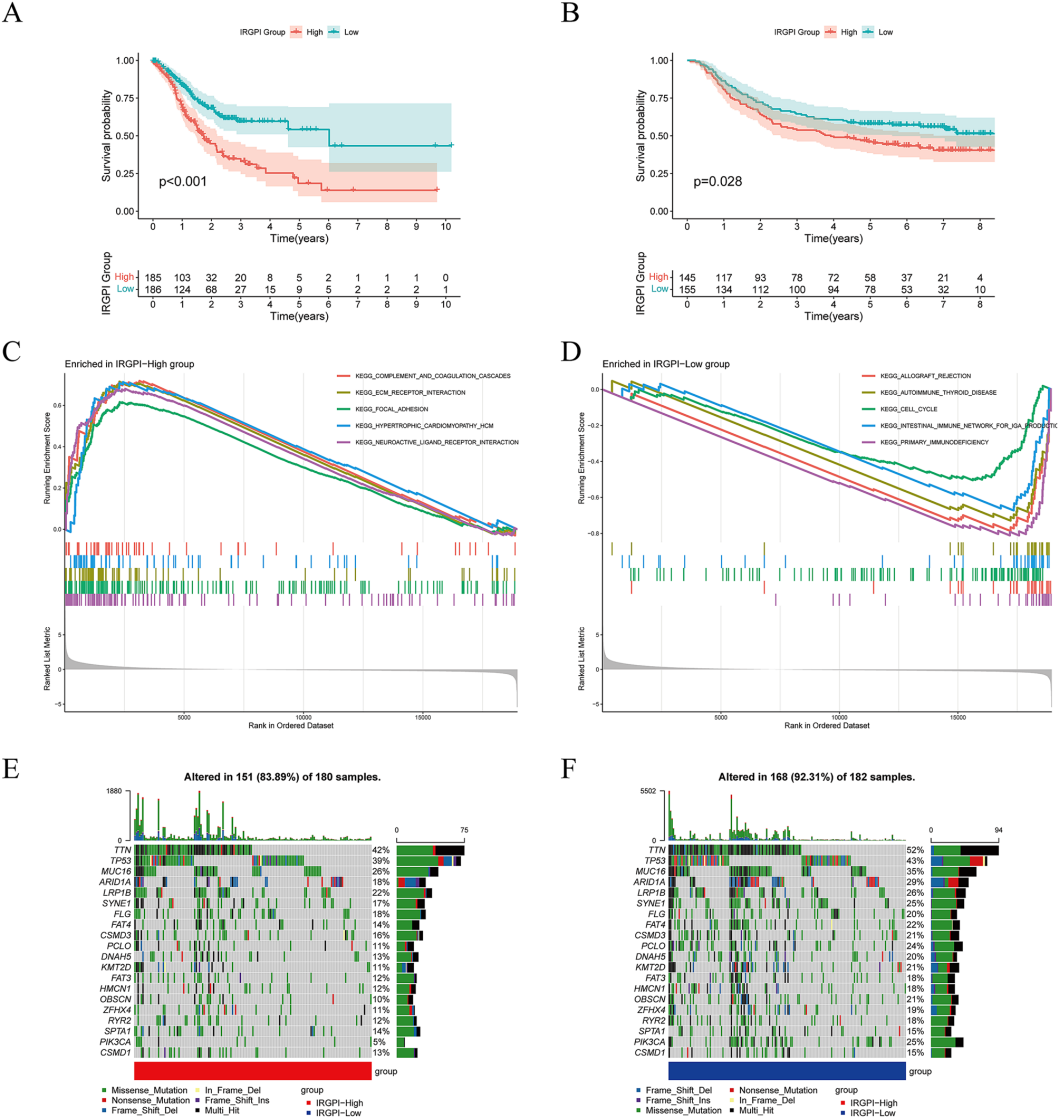
Prognostic analysis and molecular characteristics of different IRGPI groups. (**A**) Kaplan–Meier survival analysis of the IRGPI groups in the TCGA cohort. (**B**) Kaplan–Meier survival analysis of the IRGPI groups in the GEO cohort. (**C**) Gene sets enriched in IRGPI-High group in TCGA-STAD cohort. (**D**) Gene sets enriched in IRGPI-Low group in TCGA-STAD cohort. (**E**) Gene mutation landscape in IRGPI-High group in TCGA-STAD cohort. (**F**) Gene mutation landscape in IRGPI-Low group in TCGA-STAD cohort. [Figures created by R, version 4.0.4.].

Patients with IRGPI-low had superior OS than those with IRGPI-high (p < 0.001, log-rank test) when the median IRGPI was used as the threshold value in the TCGA-STAD cohort (Fig. 3A). Moreover, we performed survival analysis on the GSE62254 (n = 300) GC dataset to assess the generality of IRGPI in prognosis. Equally, patients in the IRGPI-low group fared better than those in the high IRGPI group (p = 0.028, log-rank test) (Fig. 3B).

GSEA was utilized to identify the set of genes that were enriched in distinct IRGPI groups in TCGA-STAD (Fig. 3C,D). The IRGPI-high group was significantly enriched in ECM receptor interactions and focal adhesion pathways that are associated with metastasis with cancer. This suggests that the IRGPI-high group is more prone to tumor metastasis and that patients have a poor prognosis. The IRGPI-low group was enriched in DNA replication and immune-related pathways, suggesting that the IRGPI-low group had better anti-tumor immunity, demonstrating the good prognosis of GC patients. Furthermore, we validated the enrichment in the GSE64425 cohort. As shown in the Fig. S3A, we obtained enrichment results in the GSE64425 cohort consistent with the TCGA-STAD cohort. The detailed results of GSEA are listed in Table S2.

Gene mutations contribute to further biological insight into the tumorigenesis in GC, thereby we explored the immunological nature of the IRGPI group by analyzing gene mutations. In the TCGA-STAD cohort, we found a high number of mutations in both the group with IRGPI-high and the group with IRGPI-low. The most prevalent form of mutation was a missense variation. The top 20 genes in the IRGPI group with the highest mutation rates were then found. TTN, TP53, MUC16, ARID1A, LRP1B, SYNE1, and FLG mutation rates were greater than 15% in both groups (Fig. 3E,F).

### Relationship between IRGPI grouping and immunological subtypes

**Figure 4:**
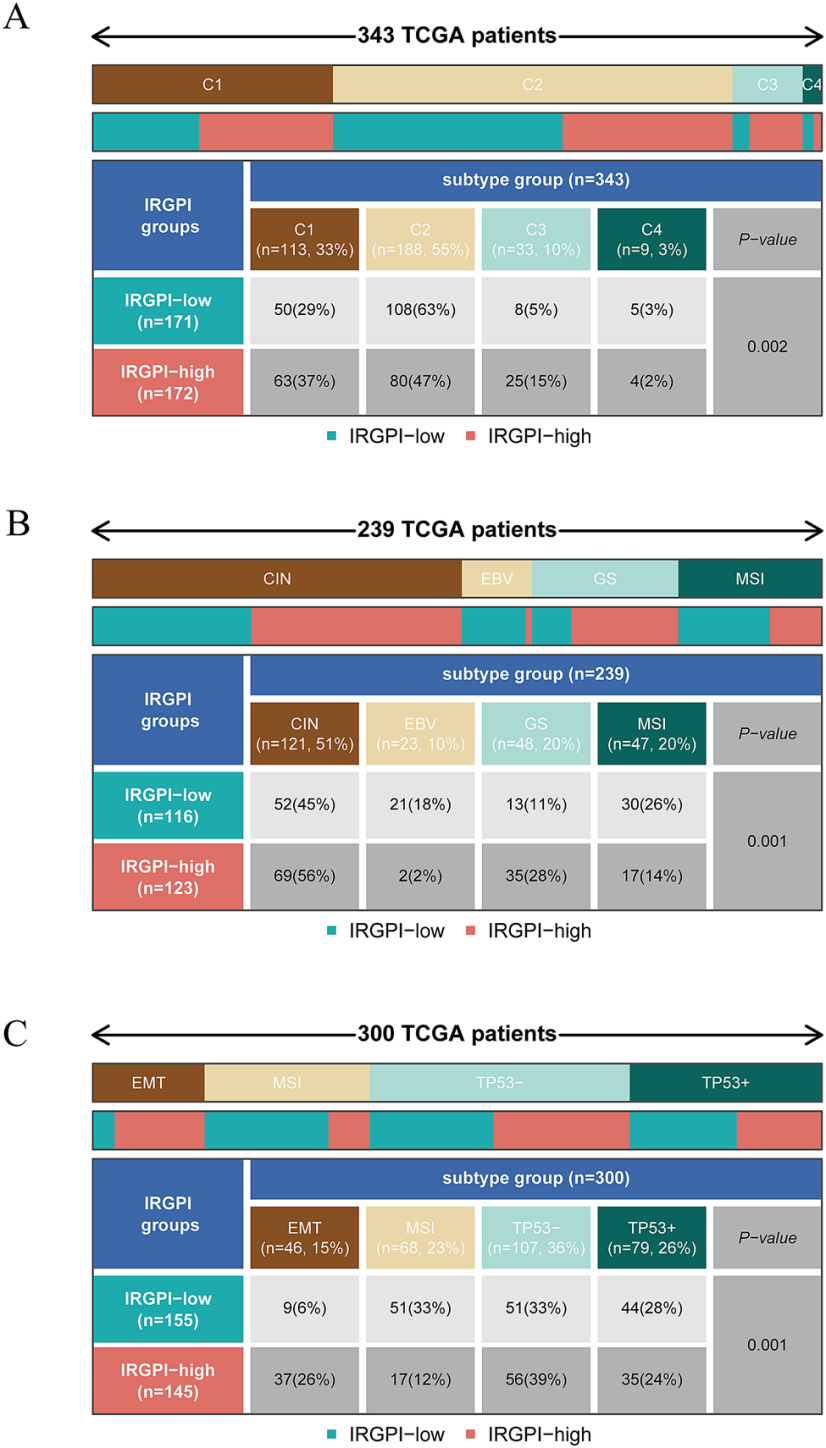
The distribution of immune and molecular subtypes in different IRGPI groups. (**A**) The distribution of TCGA immune subtypes between IRGPI groups in TCGA-STAD cohort. (**B**) The distribution of molecular subtypes between IRGPI groups in TCGA-STAD cohort. (**C**) The distribution of ACRG subtypes between IRGPI groups in GSE62254 cohort. [Figures created by R, version 4.0.4.].

Immune subtypes determine a tumor's immune state depending on the tumor and stromal compartment. A consensus immune subtype summarizes six subtypes^27^. The C1 subtype is represented by wound healing with high expression of angiogenic genes as well as adaptive immune infiltration. The C2 subtype is characterized by IFN-γ dominance with the highest M1/M2 macrophage polarization and strong CD8 signaling. Then, we focused on the distribution of immune subtypes in the IRGPI group. As illustrated in Fig. 4A, in TCGA-STAD cohort, the C2 subtype was more prevalent in the IRGPI-low group, while the C1 subtype was more frequent in the IRGPI-high group (p = 0.002, chi-square test).

The Cancer Genome Atlas (TCGA) conducted a thorough molecular analysis of GC and proposed a new molecular subtype of GC into four subtypes: CIN, EBV, GS, and MSI^28^. GS subtypes exhibited elevated expression of cell adhesion pathways, angiogenesis-related pathways, and pathways associated with syndecan-1-mediated signaling. The distribution of molecular subtypes in the IRGPI group was next evaluated. In our research (Fig. 4B), the IRGPI-low group had greater MSI and lower GS than the IRGPI-high group in TCGA-STAD cohort. (p = 0.001, chi-square test).

The Asian Cancer Research Group (ACRG), used gene expression data to characterize four molecular isoforms associated with different patterns of molecular alterations, disease progression, and prognosis^17^. In our study (Fig. 4C), MSI subtypes were found to be more common in the IRGPI-low group, while EMT subtypes were more prevalent in the IRGPI-high group in the GSE62254 cohort. (p = 0.001, chi-square test).

### Immune characteristics of different IRGPI groups

**Figure 5:**
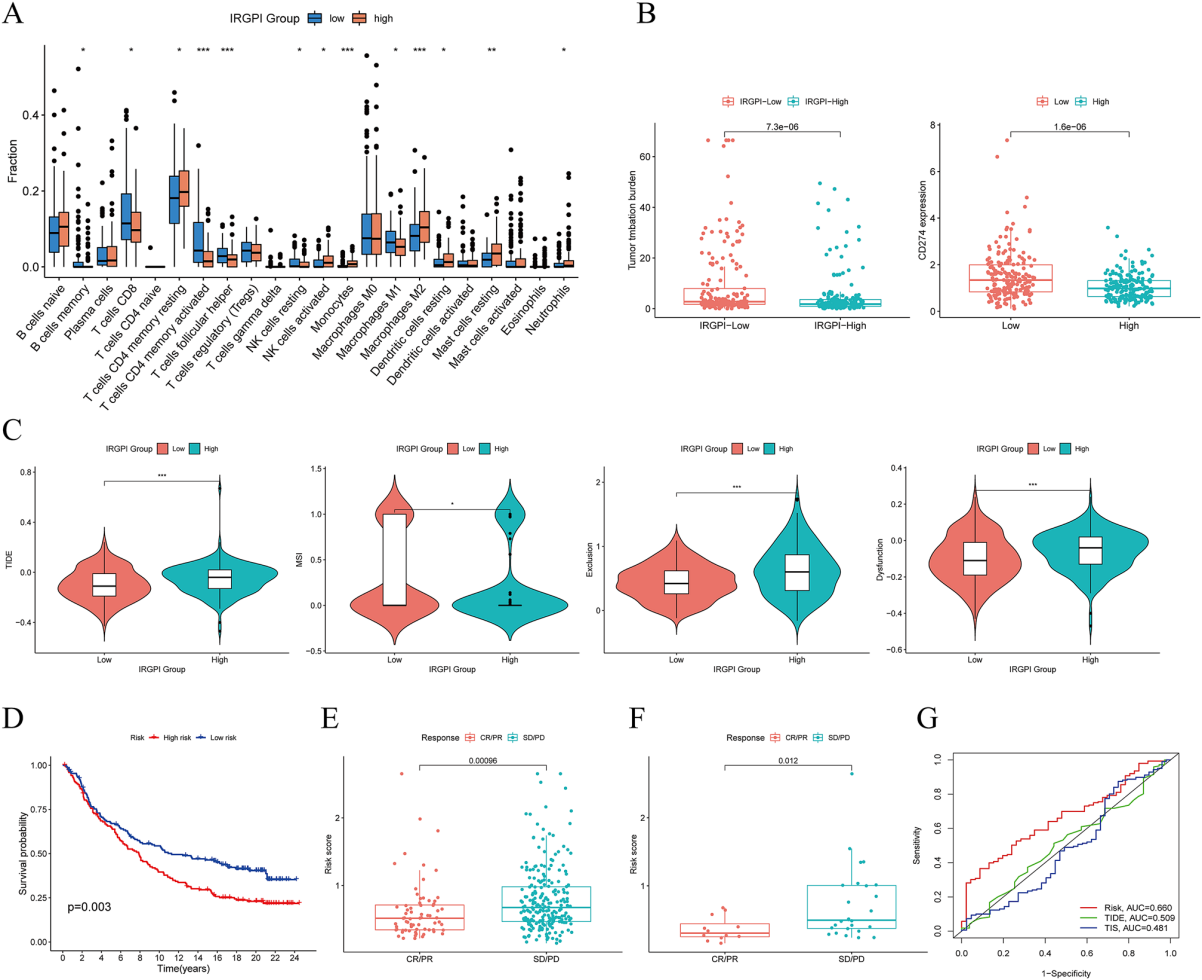
Prognostic value of IRGPI in immunotherapy. (**A**) The proportions of immune cells in different IRGPI groups. (**B**) Comparison of TMB and PD-L1 expression in different IRGPI groups. (**C**) TIDE, MSI, and T cell exclusion and dysfunction score in different IRGPI groups. (**D**) Kaplan–Meier survival analysis of the IRGPI groups in the IMvigor210 cohort. (**E**) IMvigor210 cohort of CR/PR and SD/PD in different IRGPI groups. (**F**) PRJEB25780 cohort of CR/PR and SD/PD in different IRGPI groups. (**G**) ROC analysis of IRGPI, TIS, and TIDE on overall survival at 1-, 3-, and 5-years OS in TCGA-STAD cohort. [Figures created by R, version 4.0.4.].

To investigate the differences in the tumor microenvironment (TME) by analyzing the distribution of immune cells in various IRGPI groups (Table S3). In TCGA-STAD cohort, the IRGPI-high group had more monocytes, M2 macrophages, and neutrophils, whereas the IRGPI-low group had more CD8 T cells, activated memory CD4 T cells, follicular helper T cells, and M1 macrophages (Fig. 5A). Equally, we found the same conclusion for the GSE62254 cohort (Fig. S3B). Following that, we evaluated the association between IRGPI score, PD-L1 expression, and TMB in the TCGA-STAD cohort. Figure 5B shows the results, which reveal that the IRGPI score was associated significantly with TMB(p = 7.3e−06) and the IRGPI score was related to PD-L1 expression (p = 1.6e−06). Prospective studies have shown that TMB is a potential biomarker for predicting response to ICI therapy. the higher the TMB level, the better the outcome of patients receiving ICI therapy. Tumors with high PD-L1 expression responded better to anti-PD-L1 therapy than those with low PD-L1 expression. This is consistent with the results obtained in our study.

### The benefits of ICI therapy in various IRGPI groups

TIDE was utilized to evaluate the potential clinical effectiveness of immunotherapy in different IRGPI groups. The greater TIDE score indicates a higher risk of immune evasion, implying that these individuals might less benefit from ICI therapy^21^. According to our findings (Table S4), in TCGA-STAD cohort, patients with IRGPI-low had a lower TIDE score than those with IRGPI-high, showing that patients with IRGPI-low might gain more from ICI therapy (Fig. 5C). We assessed the prognostic value of IRGPI in IMvigor210 patients receiving anti-PD-L1 therapy^29^ (Fig. 5D). We also compared the treatment response of patients in the advanced gastric cancer cohort PRJEB25780 who received PD-L1 therapy (Fig. 5E,F). Surprisingly, it was found that lower IRGPI scores had higher immunotherapy effects. Furthermore, we compared the prediction capability of IRGPI to TIDE and TIS scores and determined that the reliability of IRGPI was greater than TIDE and TIS^21,26^ (Fig. 5G). These findings suggested that the IRGPI could be a viable biomarker for predicting ICI therapy response.

## Discussion

Since the overall response rate to ICI remains low, it is crucial to determine which patients could benefit from it. Identifying an accurate biomarker to predict response to immunotherapy remains a crucial problem in the development of ICI for gastric cancer. To develop a predictive index based on immune-related differentially expressed genes in GC, we utilized WGCNA and regression analysis to determine five immune-related hub genes. We used weighted gene expression levels to calculate the IRGPI score and validated it as an independent and valid prognostic factor. In both the TCGA and GEO cohorts, the IRGPI was demonstrated to be a meaningful predictive immune-related biomarker for GC prognosis, with better survival in IRGPI-low patients and worse survival in IRGPI-high patients.

To gain insight into the molecular characterization, we investigated the differences in gene mutations between the IRGPI groups and found significant differences in TTN, TP53, and MUC16 gene mutations. Furthermore, the IRGPI-low group had a higher rate of MUC16 gene mutations than the IRGPI-high group (35 vs. 25%). In the pan-cancer dataset, MUC16 and TTN mutations each showed significantly better OS^30^. MUC16 mutations may predict a better prognosis for GC patients^31^. MUC16 knockdown hindered PI3K/Akt/mTOR signaling and reduced the protein level of Myc, a crucial transcription factor that controls glycolysis^32^. Thus, IRGPI-low patients with high TTN and MUC16 mutations had better outcomes than patients with IRGPI-high patients with low TTN and MUC16 mutations, consistent with our survival results.

Understanding TME is of crucial value in improving the effectiveness of immunotherapy. The distribution of immune cells in cancer tissues differed significantly between the two IRGPI groups. M2 macrophages and neutrophils were discovered to be more abundant in the IRGPI-high group, whereas CD8 T cells, activated memory CD4 T cells, M1 macrophages, and follicular helper T cells were found to be more abundant in the IRGPI-low group. Neutrophils were shown to be abundant in the GC environment, which was related to tumor growth and poor patient survival. Neutrophils strongly upregulate CD54 and B7-H4 expression in the GC microenvironment, and B7-H4-mediated antitumor immunosuppression is one of the main mechanisms driving T cell dysfunction^33^. In a majority of tumors, the major subtype of macrophages is the M2 macrophage, which has been linked to chronic inflammation that favors tumor progression and the formation of an infiltrative phenotype, and which is connected with poor prognosis in gastric, breast, and prostate cancers. In contrast, high-density M1 macrophage infiltration could be related to acute inflammation and predict a positive prognosis in patients with HCC, NSCLC, or gastric cancers^5,34,35^. Numerous studies have demonstrated that a significant concentration of T cell infiltration, particularly cytotoxic CD8T cells, predicts a favorable prognosis^3,36^. Our findings support these conclusions.

Next, we discuss the relationship between IRGPI and known predictive biomarkers for immunotherapy, such as PD-L1 and TMB^37–39^. We found the correlation between IRGPI score, PD-L1 expression, and TMB, which helps to explain how IRGPI affects the prognosis of immunotherapy. TIDE is a novel calculation that has shown predictive power in different solid tumors. TIDE scores correlate with T cell dysfunction in cancers with high CTL and T cell exclusion in tumors with low CTL, indicating two distinct immune evasion strategies^21^. In our study, IRGPI-high patients displayed lower CTL penetration and higher TIDE, T cell exclusion, and T-cell dysfunction scores than IRGPI-low patients, indicating that immune evasion through T cell exclusion was the primary cause of their reduced ICI response. The IRGPI-low group, on the other hand, performed a higher MSI score and lower TIDE score than the IRGPI-high group, suggesting that these patients had low levels of immune evasion and greater MSI. The beneficial effect of MSI on immunotherapy for GC has been demonstrated, with MSI resulting in a high mutational burden that renders the tumor immunogenic and responsive to anti-PD1 treatment^40–42^. We performed a survival analysis of the IMvigor210 cohort treated with anti-PD-L1 therapy to ascertain the prognostic utility of IRGPI. It was found that patients with low IRGPI scores had better immunotherapy responses in both IMvigor210 and PRJEB25780 cohorts. Tumor Inflammatory Signaling (TIS) is an 18-gene signature that indicates genes for sustained adaptive Th1 and cytotoxic CD8+ T cell responses and has shown promising results in predicting response to anti-PD-1/PD-L1 drugs^26^. However, both TIDE and TIS focused on patient response to immunotherapy. In our study, the predictive value of IRGPI was comparable to that of TIDE and TIS, and IRGPI could better predict OS. In summary, IRGPI is a potential immune-related prognostic biomarker that could predict the efficiency of ICI therapy as well as the overall survival of GC patients. IRGPI grouping could help to distinguish tumor immune microenvironment and molecular features, but further studies are required to clarify this point.

## Supplementary Information

Supplementary Information 1.

Supplementary Information 2.

## Data Availability

The databases used in this study are all publicly available and can be found in the TCGA database (https://portal.gdc.cancer.gov/) and the GEO (https://www.ncbi.nlm.nih.gov/geo/) database.
