# Supplementary material for: Development and verification of an immune-related gene prognostic index for gastric cancer
Source: Sci Rep. 2022 Sep 20;12:15693. doi: 10.1038/s41598-022-20007-y (PMC9489759; doi:10.1038/s41598-022-20007-y)
Supplement: Supplementary file 1 — Supplementary Information 1. [file 41598_2022_20007_MOESM1_ESM.pdf]

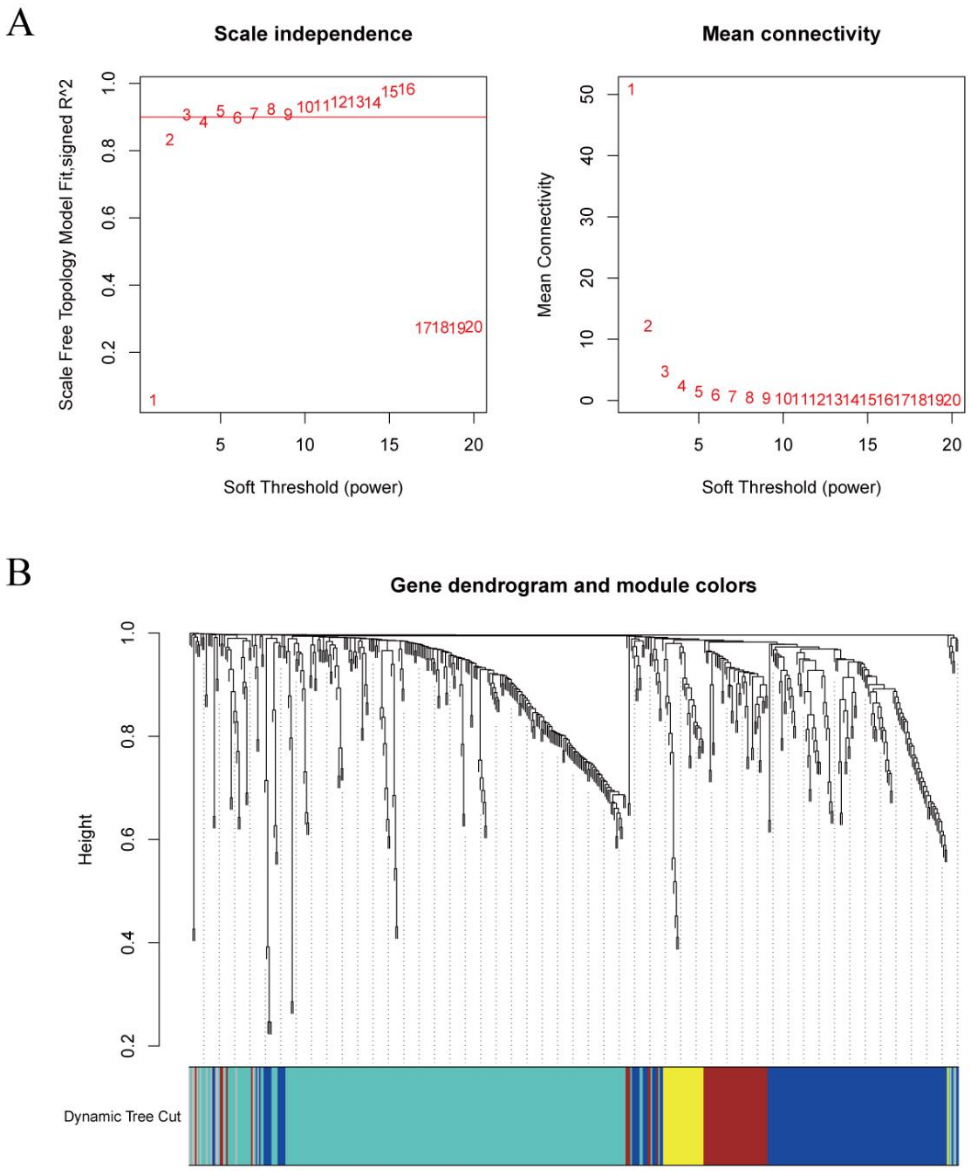

Fig S1 (A) The scale-free fit index and mean connectivity for various soft-thresholding powers. (**B**) Module identification based on dynamic pruning tree. [Figures created by R, version 4.0.4.]

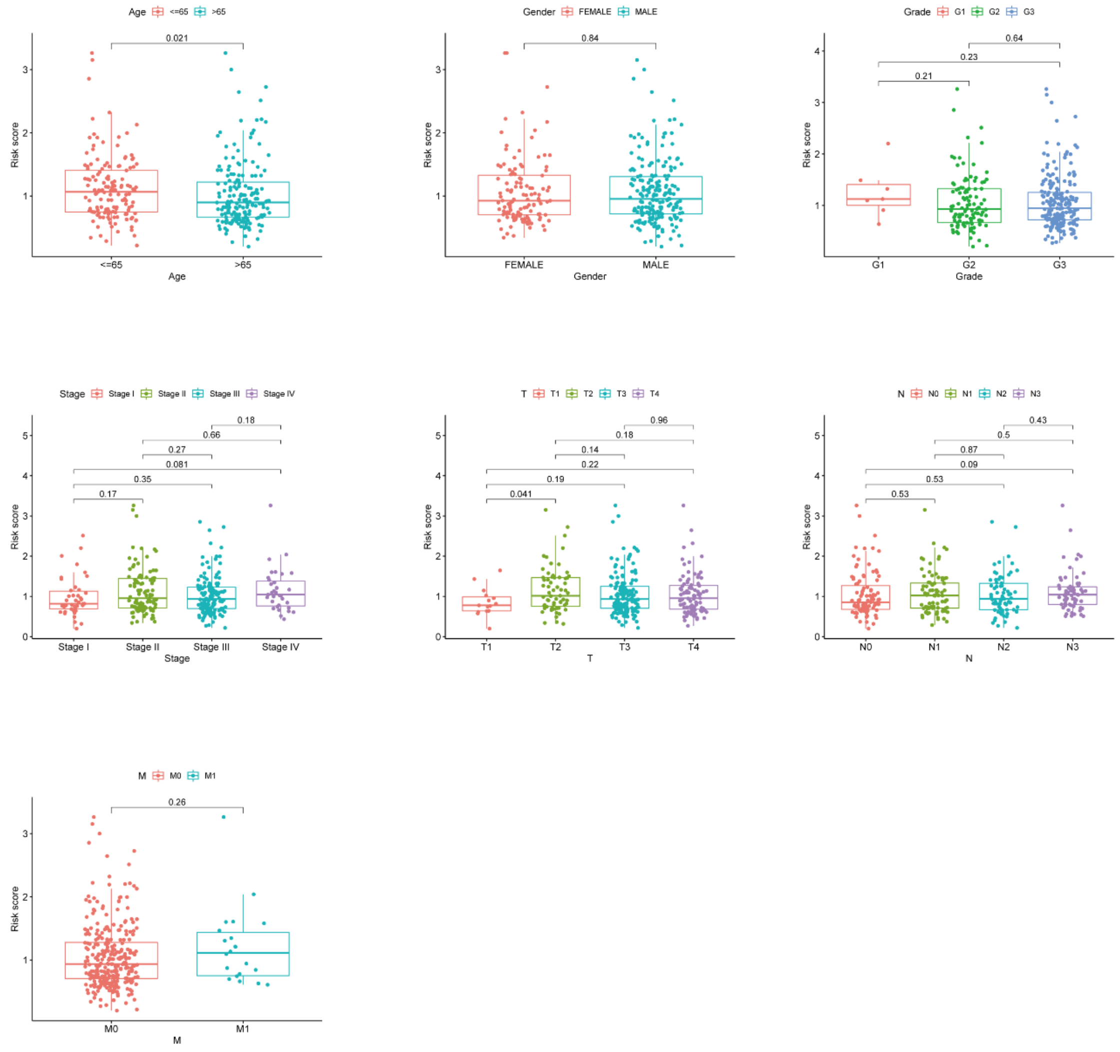

Fig. S2 Correlation between clinical characteristics and IRGPI. [Figures created by R, version 4.0.4.]

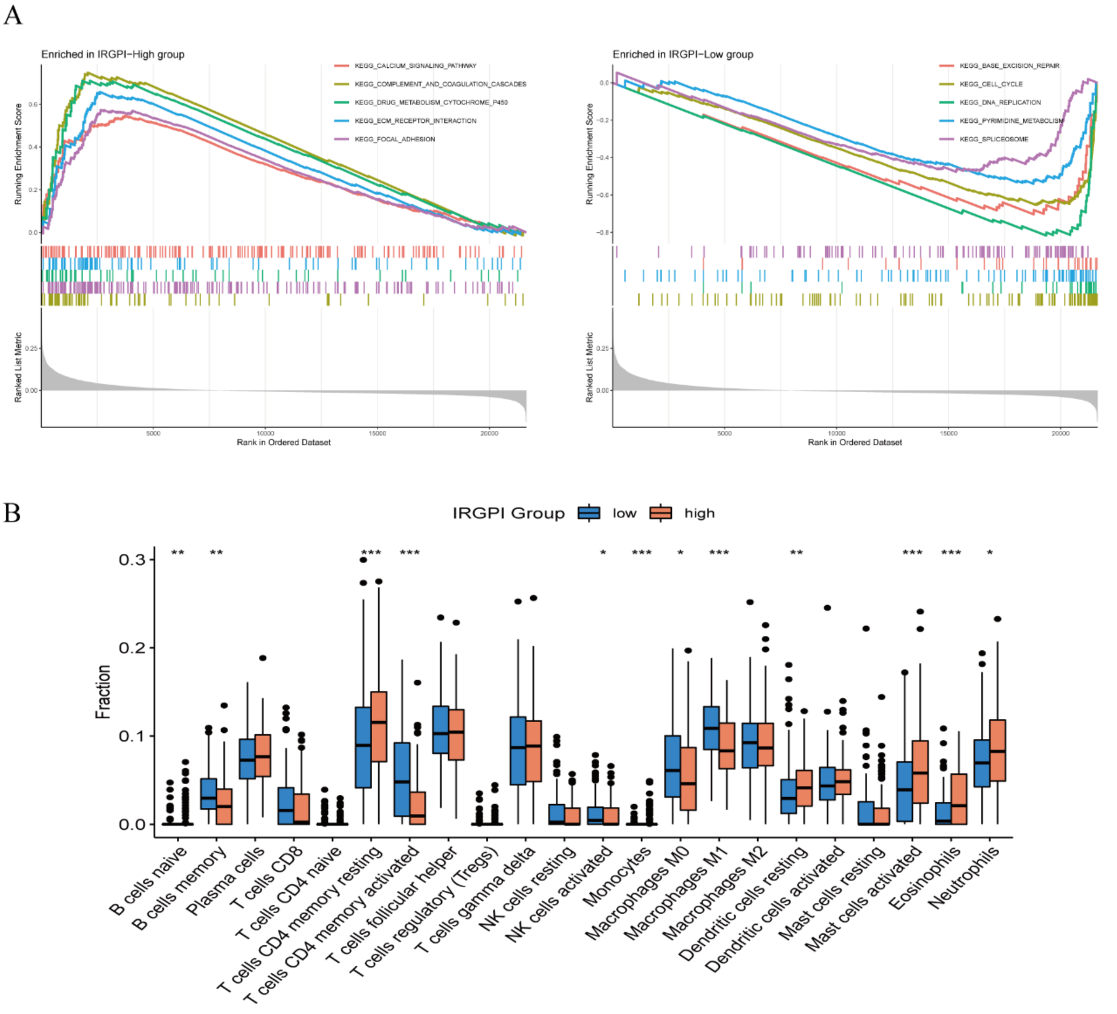

Fig S3 Biological pathways and immune cell infiltration validated in the GSE62254 cohort (A) Gene sets enriched in IRGPI-High and IRGPI-Low groups in the GSE62254 cohort. (B) The proportions of TME cells in different IRGPI groups in the GSE62254 cohort. [Figures created by R, version 4.0.4.]
